# Increased plasma levels of circulating cell-free mitochondrial DNA in suicide attempters: associations with HPA-axis hyperactivity

**DOI:** 10.1038/tp.2016.236

**Published:** 2016-12-06

**Authors:** D Lindqvist, J Fernström, C Grudet, L Ljunggren, L Träskman-Bendz, L Ohlsson, Å Westrin

**Affiliations:** 1 Department of Clinical Sciences Lund, Psychiatry, Lund University, Lund, Sweden; 2 Department of Psychiatry, School of Medicine, University of California San Francisco, School of Medicine, San Francisco, CA, USA; 3 Psychiatric Clinic, Lund, Division of Psychiatry, Lund, Sweden; 4 Department of Biomedical Science, Malmö University,Health and Society, Malmö, Sweden

## Abstract

Preclinical data suggest that chronic stress may cause cellular damage and mitochondrial dysfunction, potentially leading to the release of mitochondrial DNA (mtDNA) into the bloodstream. Major depressive disorder has been associated with an increased amount of mtDNA in leukocytes from saliva samples and blood; however, no previous studies have measured plasma levels of free-circulating mtDNA in a clinical psychiatric sample. In this study, free circulating mtDNA was quantified in plasma samples from 37 suicide attempters, who had undergone a dexamethasone suppression test (DST), and 37 healthy controls. We hypothesized that free circulating mtDNA would be elevated in the suicide attempters and would be associated with hypothalamic–pituitary–adrenal (HPA)-axis hyperactivity. Suicide attempters had significantly higher plasma levels of free-circulating mtDNA compared with healthy controls at different time points (pre- and post-DST; all *P*-values<2.98E−12, Cohen's *d* ranging from 2.55 to 4.01). Pre-DST plasma levels of mtDNA were positively correlated with post-DST cortisol levels (rho=0.49, *P*<0.003). Suicide attempters may have elevated plasma levels of free-circulating mtDNA, which are related to impaired HPA-axis negative feedback. This peripheral index is consistent with an increased cellular or mitochondrial damage. The specific cells and tissues contributing to plasma levels of free-circulating mtDNA are not known, as is the specificity of this finding for suicide attempters. Future studies are needed in order to better understand the relevance of increased free-circulating mtDNA in relation to the pathophysiology underlying suicidal behavior and depression.

## Introduction

Mitochondria are cytoplasmic organelles of the eukaryotic cells that have many important roles in cellular function. Mitochondria can be conceptualized as the ‘power generators' of the cell, utilizing oxygen, energy substrates (carbohydrates and lipids) and other compounds to form energy-rich phosphates.^1, 2^ Furthermore mitochondria are also involved in other important cellular processes, such as apoptotic and necrotic cell death,^1, 3^ the generation and regulation of free radicals,^4, 5, 6^ gene expression regulation^7, 8^ as well as signal transduction for cell proliferation and differentiation.^1, 3^ Each cell contains multiple mitochondria, and each mitochondrion possesses its own genome; the maternally inherited mitochondrial DNA (mtDNA) encoding for 37 different genes involved in energy production.^9, 10, 11^ MtDNA is thought to be highly susceptible to oxidative damage due to limited DNA repair mechanisms.^12^ Moreover, chronic stress may alter mitochondrial structure and function via increased levels of glucocorticoids, the primary mediators of the stress response.^13^ Glucocorticoid levels seem to regulate mitochondrial function in neurons in an inverted U-shaped manner. Du *et al.*^14^ showed, in a series of *in vivo* and *in vitro* experiments, that physiological doses of glucocorticoids increased the mitochondrial membrane potential and resistance to apoptosis, whereas long-lasting and higher doses of glucocorticoids were associated with decreased mitochondrial membrane potential and increased sensitivity for apoptosis. Furthermore, animal studies have shown that chronic stress exposure leads to mitochondrial damage and dysfunction in various brain regions such as the hippocampus, cortex and thalamus.^15, 16, 17^ Yet another experimental study demonstrated that dexamethasone (a cortisol analog) may cause mitochondrial fragmentation in hepatocytes via the induction of proteins that promote mitochondrial fission.^18^ Mitochondrial fragmentation, in turn, activates pro-apoptotic mechanisms that sensitize the cell toward programmed cell death.^19^

Although the exact mechanisms are still unclear, dysfunctional mitochondria, perhaps due to chronic stress, are thought to promote the release of mtDNA into the circulation. Free circulating mtDNA may activate several deleterious downstream mechanisms including systemic inflammation,^13^ potentially causing depressive symptoms; however, no previous studies have measured plasma concentrations of free-circulating mtDNA in a psychiatric sample. In their seminal study, Cai *et al.* found that major depressive disorder (MDD) was associated with greater amount of mtDNA in leukocytes from saliva samples and blood.^20^ Moreover, higher levels of mtDNA were (i) significantly associated with stressful life events in humans and (ii) were associated with a stress paradigm and corticosteroid administration in animals.^20^ Some of the same authors subsequently confirmed, in a longitudinal study, that high leukocyte copy numbers of mtDNA (mtDNA-cn) was associated with mood disorders.^21^ The difference in leukocyte mtDNA-cn between MDD cases and controls was most significant at week 6 of the depressive episode; however, mtDNA-cn did not correlate with severity of depressive symptoms.^21^

The present study sought to determine whether free-circulating mtDNA in cell-free plasma is increased in suicide attempters, a group often characterized by depressive symptoms, increased psychological stress and adverse childhood events.^22^ On the basis of previous animal experiments, we also wanted to test the relationship between free-circulating plasma levels of mtDNA and hypothalamic–pituitary–adrenal (HPA)-axis activity in this sample. For this purpose, we measured free-circulating mtDNA in plasma samples from 37 recent suicide attempters, who had undergone a dexamethasone suppression test (DST), and 37 healthy controls. We hypothesized that plasma levels of free-circulating mtDNA would be significantly higher in the suicide attempters and that higher levels would be associated with a dysregulated stress response.

## Materials and methods

### Ethical approval

The study was approved by the Lund University Medical Ethics Commitee, and all patients gave informed consent to participate in the study.

### Subject recruitment, suicide attempters

Between 1992 and 2001, patients with a recent suicide attempt, as defined by Beck *et al.*,^23^ were proposed to participate in a research project that has been described in more detail elsewhere.^24, 25^ Suicide attempters were evaluated by a certified psychiatrist for DSM axis I and II diagnoses.^26^

The Montgomery-Åsberg Depression Rating Scale (MADRS)^27^ was used to assess depressive symptoms. Suicide attempters did not receive any antidepressants or antipsychotics during a washout period (mean±s.d.: 12±13 days) before blood sampling and the DST. Occasional doses of benzodiazepines were allowed during the washout.

In total, 37 plasma samples from medication-free subjects who had undergone the DST were selected from the above-mentioned larger study. The rationale for selecting cases that had undergone the DST was that the aim of the study, in addition to investigate the level of free-circulating mtDNA in cases and controls, was to test the association between concentration of mtDNA in cell-free plasma and post-DST cortisol levels in suicide attempters. The plasma samples from the patients had not been thawed before the analysis of mtDNA. For the control group, all plasma samples (with the exception of one unthawed sample) had been thawed and frozen once before analysis.

Twenty-nine of the included suicide attempters in this study had taken a drug overdose, four subjects had attempted to hang themselves, two subjects had used wrist-cutting and two subjects had taken a drug overdose in combination with wrist-cutting. None of the suicide attempters had a medical condition or took any medications (such as corticosteroids or insulin) known to interfere with the results of the DST. After the washout period, drug screening showed no traces of antidepressants or antipsychotics in the included subjects, but five subjects showed traces of benzodiazepines.

### Subject recruitment, controls

Thirty-seven healthy controls were recruited in 1994. Healthy controls were employees or students at the Lund University Hospital or retirees. They were all medically examined by one of the co-authors (ÅW). As determined by semi-structured interviews, physical examination and blood analysis, none of the controls had any prior or current psychiatric disorders or any somatic disorders (including endocrine, nervous, hepatic, renal, cardiac, asthmatic, hypertensive or infective disorders). None of the controls took any corticosteroids, insulin or other medications that could interfere with the DST.

### DST and sample preparation

One mg dexamethasone was given at 2200 hours, and blood samples were drawn, in either serum- or EDTA tubes, at 1500 hours on the day of dexamethasone administration, as well as at 0800 and 1500 hours the following day.^28^ Serum tubes were left at room temperature for 30 min before centrifugation, and plasma tubes were immediately placed on ice until centrifugation. Blood samples were centrifuged at 4 °C and 2000 *g* for 10 min within 1 h of blood collection. Plasma was stored in −70 °C until analysis of mtDNA. At all three time points, cortisol was analyzed in serum (suicide attempters only) and free-circulating mtDNA was analyzed in plasma (in all subjects). Serum analysis of cortisol was performed either on the same day or stored at −20 °C until analysis. Serum cortisol was measured using a commercial radioimmunoassay (RIA) (Orion diagnostica RIA kit). The detection limit was 7 nmol l^−1^, and the intra- and interassay coefficients of variation were 5% and 7%, respectively.

### Measurement of free-circulating mtDNA

DNA was isolated from thawed plasma samples using the QIAmp 96 DNA Blood Kit (Qiagen, Valencia, CA, USA) according to the manufacturer's instruction for Blood and body-fluid protocol. Before the isolation of DNA, the plasma samples were centrifugated at 10 000 *g* for 10 min. The purity of the eluted DNA was measured using spectrophotometric analysis at 260/280 nm in a Nanodrop (ND-1000 Spectrophotometer v 3.7.1, Waltham, MA, USA).

The quantitative analysis of cell-free mtDNA was performed using quantitative real time polymerase chain reaction (PCR). The experiment was run once in triplicate reactions. A dilution series consisting of the PCR product was constructed and used to create a standard curve. The different crossing-point values from the unknown samples were compared with the standard curve, and the corresponding number of mitochondrial units was calculated using the following formula:


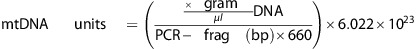


The amount of DNA (g μl^−1^) was divided with the size of the PCR fragment (bp) and the molar mass per base pair (g mol^−1^). The product was finally multiplied with Avogadro's constant.

The primers (Life Technologies, Paisley, UK) used for PCR amplification of mtDNA were as stated in the table below:
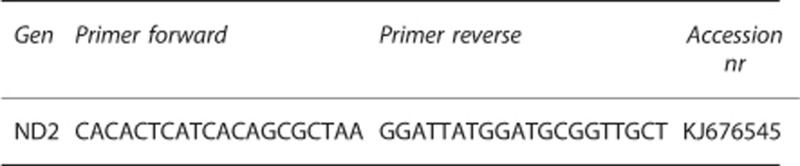


The PCR reactions were carried out using SYBR Green Technology (Thermo Fisher Scientific, Waltham, MA, USA). Each 20 μl reaction contained 5 μl of template, 1 μl of each primer (10 μM), 10 μl SYBR MIX (2 ×, Sensifast, Bioline, London, UK) and 3 μl of nuclease-free water. Each reaction was run in triplicate on a LC480 LightCycler from Roche, Mannheim, Germany) using the following program:

Initial denaturation at 95 °C for 10 min, followed by 45 cycles consisting of 95 °C in 10 s. for melting, 65 °C for 10 s annealing and 72 °C for 11 s extension. The program ended with a melting curve analysis measuring fluorescence continuously from 60 to 97 °C.

### Statistics

The Statistical Package for the Social Sciences for Mac (version 23, IBM, Armonk, NY, USA) was used for statistical calculations. The concentration of mtDNA was skewed at all three time points. Therefore, and because some of the group-wise comparisons involved smaller subgroups, Mann–Whitney *U-*test was used to test differences in mtDNA concentration across groups. Spearman's Rho was used for correlations involving mtDNA level. Student's *t*-test was used to compare age and body mass index (BMI) between groups and Pearson's *χ*^2^ was used to compare sex distribution across groups. Effect size was calculated using Cohen's *d* on log-transformed data. Cohen's *d*>0.2 is generally considered a small effect size, >0.5 medium and >0.8 large. All tests were two-tailed. For correlations between mtDNA units and post-DST cortisol levels, we carried out a total number of six tests (each of the three mtDNA measurements versus each of the two post-DST cortisol measurements); thus, the Bonferroni-adjusted *P*-value for the correlation analyses was set to 0.008 (0.05/6). Within the suicide attempters, we compared the mtDNA levels between repeaters versus non-repeaters, violent versus non-violent suicide attempters and suicide attempters with MDD diagnosis versus suicide attempters without MDD diagnosis. For these three subgroup-analyses, the Bonferroni-adjusted *P*-value was set to 0.017 (0.05/3).

## Results

### Demographics

Demographic and clinical characteristics of all subjects are presented in Table 1. Groups were balanced in terms of age, sex and BMI. Within the suicide attempter group, free-circulating mtDNA (measured at any of the three time points) was not significantly correlated with number of washout days or number of years stored in the freezer (all *P*>0.57).

Eighteen of the cases had attempted suicide more than once (‘repeaters') and seven had used a violent suicide method. Drug overdoses, single wrist-cutting or a combination of both are considered non-violent suicide attempts, whereas all other methods (for example, hanging, use of firearms or several deep knife cuts) are classified as violent.^29^ For 14 cases, MDD was the principal axis 1 diagnosis, five cases had dysthymic disorder, six cases had Adjustment Disorder, five cases Depression Not Otherwise Specified, one case Anorexia Nervosa and six cases did not meet the criteria for an axis 1 disorder. Twenty of the cases had an axis II personality disorder, cluster B being the most frequent specifier (*n*=9). MADRS scores were available from 31 of the suicide attempters and did not correlate significantly with mtDNA units at any of the three time points (*P*>0.29).

### mtDNA units versus clinical variables

**Figure 1:**
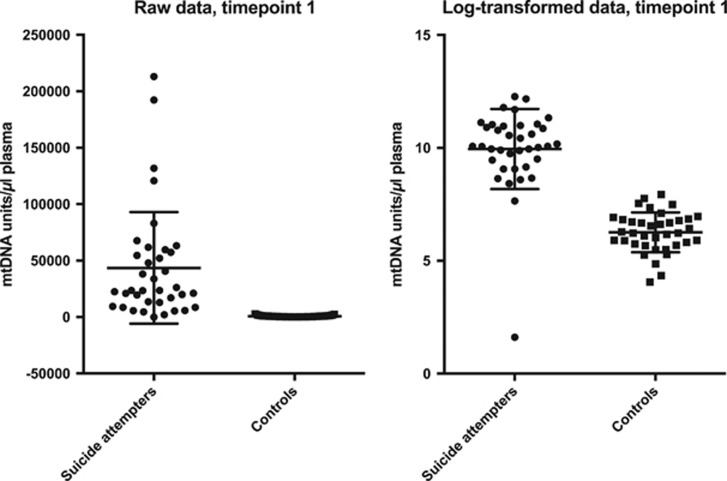
Free-circulating plasma mtDNA units in suicide attempters and controls at time point 1 (raw and log-transformed data, respectively). Error bars indicate 1±s.d. from the mean. mtDNA, mitochondrial DNA.

**Figure 2:**
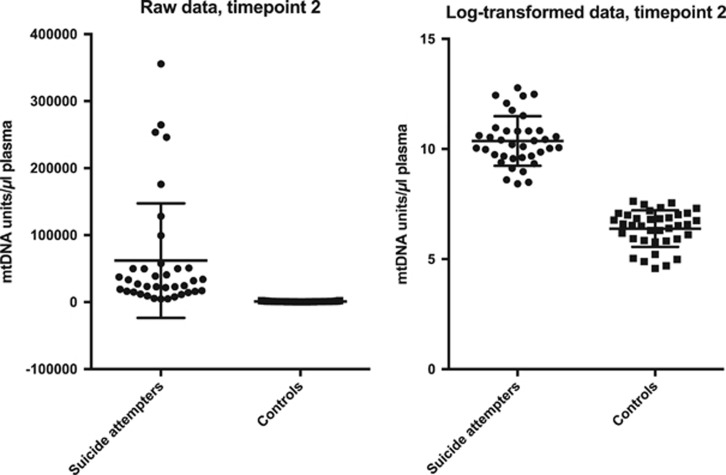
Free-circulating plasma mtDNA units in suicide attempters and controls at time point 2 (raw and log-transformed data, respectively). Error bars indicate 1±s.d. from the mean. mtDNA, mitochondrial DNA.

**Figure 3:**
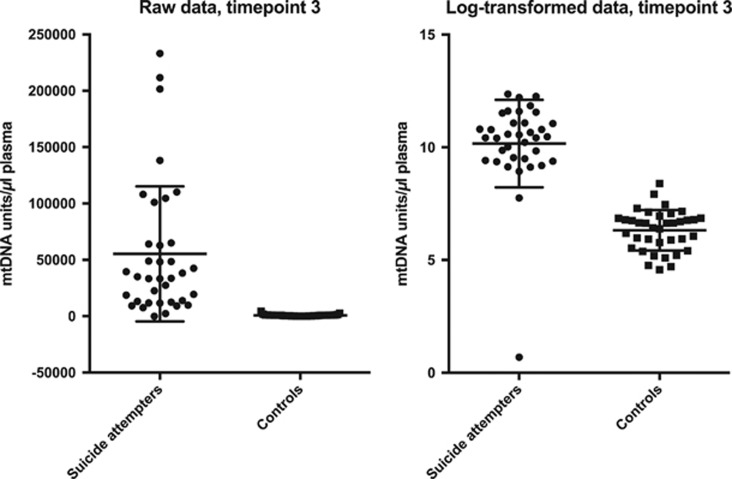
Free-circulating plasma mtDNA units in suicide attempters and controls at time point 3 (raw and log-transformed data, respectively). Error bars indicate 1±s.d. from the mean. mtDNA, mitochondrial DNA.

Suicide attempters had significantly higher plasma levels of free-circulating mtDNA compared with controls at all three time points (all *P*-values <2.98E−12, Cohen's *d* ranging from 2.55 to 4.01; Table 1). Raw and log-transformed data of free-circulating mtDNA units at all three time points in cases and controls are given in Figures 1, 2 and 3. There was no significant difference in mtDNA levels between either suicide attempters with and without MDD, violent and non-violent suicide attempters or between repeaters and non-repeaters (NS, data not shown).

### Correlations between mtDNA units and post-DST cortisol levels in suicide attempters

Correlations between mtDNA units and post-DST cortisol levels are summarized in Table 2. MtDNA units at time point 1 correlated significantly with post-dexamethasone cortisol levels at 0800 hours (rho=0.49, *P*<0.003). None of the other correlations were significant.

## Discussion

We here report that a group of medication-free suicide attempters display significantly higher plasma levels of free-circulating mtDNA compared with a group of healthy controls. The effect sizes were very large, with almost nonoverlapping groups. Within the group of suicide attempters, higher plasma levels of free-circulating mtDNA were significantly associated with high cortisol levels after a dexamethasone challenge, a marker of HPA-axis hyperactivity. In a series of experiments, Cai *et al.*^20^ recently reported that (i) MDD was associated with high amount of mtDNA-cn in leukocytes from saliva samples and blood and (ii) increased stress and corticosteroid administration was associated with increased mtDNA-cn in an animal model. Our results are in line with this study, and extend these findings by (i) reporting a robust group difference also on cell-free plasma levels of mtDNA and (ii) showing a significant relationship between cell-free mtDNA and HPA-axis hyperactivity in a clinical sample. Moreover, we did not find a significant relationship between mtDNA levels and severity of depressive symptoms, which is also in line with a previous study on leukocyte mtDNA-cn.^21^ Moreover, we did not find any significant associations between mtDNA-cn and an MDD diagnosis, suicide repetition or a violent suicide method. This suggests that high mtDNA-cn might be associated with a suicide attempt *per se* rather than certain aspects of symptom severity within this group. Another possibility is that high mtDNA-cn might reflect more long-lasting psychopathology, a hypothesis that we could not test with our cross-sectional study design. Therefore, this needs to be investigated further in longitudinal studies, taking into account the number of lifetime depressive episodes and lifetime stressors.

Increased blood levels of free-circulating mtDNA have been shown in a number of different somatic conditions such as diabetes,^30^ cancers,^31^ trauma,^32^ myocardial infarction^33^ and sepsis;^34^ thus, it is unlikely that enhanced mtDNA levels are specific for suicide attempters. Rather, cellular aging, caused by metabolic stress, inflammatory triggers or (as in the case of suicidality) severe and long-lasting psychological stress,^35^ may be a common denominator across these conditions potentially leading to increased apoptosis and a subsequent leakage of mtDNA from the damaged cells into the bloodstream. Accelerated aging at the cellular level is a biological characteristic of several psychiatric and somatic conditions associated with a heightened stress response.^36, 37, 38^ Mental illness has been associated with an elevated risk for somatic complications and early mortality.^39^ Stress-induced accelerated cellular aging and increased mtDNA may thus be common biological mechanisms causing somatic as well as psychiatric pathologies, although this hypothesis remains to be tested. Accelerated cellular aging has been associated with HPA-axis hyperactivity in stressed individuals as well as in healthy volunteers.^40, 41^ As reviewed by Picard *et al.*,^13^ chronic neuroendocrine and metabolic stress may lead to mitochondrial dysfunction and accumulation of mtDNA damage. Activation of the HPA axis increases the demand for energy production,^42^ the primary function of the mitochondria. Owing to the mitochondria's relatively poor ability to repair its own DNA and the production of reactive oxygen species, the increased metabolic rate leads to cell damage and fragmentation of mtDNA.^13^ Downstream effects in this stress-disease cascade involve cellular dysfunction and senescence, and the release of free-circulating mtDNA into the bloodstream, which in turn may have detrimental effects on multiple organ systems via inflammatory mechanisms.^13, 43^ In the present study, we found that free circulating mtDNA levels, besides being significantly different between suicide attempters and controls, were positively correlated with cortisol levels after a dexamethasone challenge. Initially proposed as a biomarker for melancholic depression,^44^ higher cortisol levels after a DST has been reported in a variety of other psychiatric disorders (ranging from psychosis to anxiety, and even in dementia),^45^ as well as somatic disorders characterized by metabolic stress such as diabetes.^46^ This suggests that high post-DST cortisol levels might be a general marker of stress, be it of metabolic or psychological origin. Our findings may suggest that psychological stress associated with the suicidal process may leave a biological imprint in the form of increased free-circulating mtDNA.

In addition to the hypothesis that stress-related cellular aging and apoptosis leads to leakage of cell-free mtDNA into the bloodstream, there is also the possibility that intracellular mtDNA content is increased, and that this is reflected in cell-free plasma. A few psychiatric studies have examined intracellular mtDNA-cn (primarily in leukocytes) with conflicting results. Reduced mtDNA-cn has been reported in post-traumatic stress disorder^47^ and in relation to depressive symptoms among elderly community-dwelling women without MDD.^48, 49^ Higher leukocyte mtDNA-cn has been reported in autism^50^ and lifetime psychopathology in healthy people.^51^ MtDNA-cn has been found to be lower^52^ and unchanged^53^ in bipolar disorder. Although the largest study to date on MDD showed significantly higher leukocyte mtDNA-cn in MDD subjects compared with controls,^20^ other studies have produced mixed results with lower,^54^ higher^21^ or unchanged^55^ mtDNA-cn in MDD. Taken together, it is unclear whether the increase in free circulating mtDNA observed in the current study is because of cell damage and subsequent mtDNA leakage into the bloodstream, increased content of intracellular mtDNA or both. Future studies on psychiatric samples should investigate these hypotheses by testing associations between free-circulating mtDNA and leukocyte mtDNA-cn as well as markers of cellular aging such as leukocyte telomere length and telomerase activity.

A major strength of this study is that the sample comprised a clinically well-characterized, somatically healthy group of medication-free suicide attempters. Moreover, the subjects underwent an inpatient washout procedure and subsequent screening for psychotropics. The present study, however, also comes with some limitations. The cross-sectional nature of our study design precludes any conclusion regarding causality, and future longitudinal studies are warranted in order to test this hypothesis. The unexpectedly large effect sizes raise the issue of whether the observed group differences were due to unaccounted factors relating to sampling, storage or health behaviors such as physical activity. Although this cannot be completely ruled out, the groups were balanced with regards to demographic characteristics such as age, sex and BMI. Moreover, all subjects were free from medications that could potentially interfere with the biological measurements. Storage time in the freezer and number of washout days were not associated with mtDNA levels, making these two factors unlikely confounders. All samples from the control group, except for one, had previously been thawed and frozen before the present analysis. Although we cannot completely rule out that this might have an impact on our results, it is unlikely as DNA is generally reported to be a very stable molecule, and a previous study found that free-circulating mtDNA levels were significantly correlated within subjects over different thaw/freeze cycles.^56^ Moreover, the control sample that had not previously been thawed showed a value of 881 mtDNA units per μl plasma, which is in line with the control group median. Another possibility is that the group differences in free-circulating mtDNA were a consequence of a physical trauma potentially associated with the suicide attempt. However, this is unlikely as no significant difference in mtDNA between violent and non-violent suicide attempters was observed. Furthermore, in most cases, 2 weeks had passed between the attempt and blood sampling. Most physical injuries, unless very severe, will have substantially improved within this time. Finally, in the present study we used high post-DST cortisol levels as a surrogate marker for increased stress and a heightened stress response. We note, however, that some other stress-related conditions such as post-traumatic stress disorder^57^ and depression related to long-term sick leave^58^ have instead been associated with HPA-axis hyporeactivity. In some subjects this might represent a pre-existing vulnerability marker for illness rather than the consequence of prolonged stress.^58^ Despite these inconsistencies across studies, we believe that our approach is justified as several lines of evidence support the notion that high post-DST cortisol is associated with increased stress in a variety of different contexts.^45^

To the best of our knowledge, this is the first study to measure free-circulating plasma mtDNA in suicide attempters and controls. Suicide attempters showed significantly higher levels of mtDNA, compared with controls, which was associated with HPA-axis hyperactivity. This peripheral index is consistent with increased cellular or mitochondrial damage. The specific cells and tissues contributing to plasma levels of free-circulating mtDNA are not known, as is the specificity of this finding for suicide attempters. Future studies are needed in order to better understand the relevance of increased free-circulating mtDNA in relation to the pathophysiology underlying suicidal behavior and depression.

## Figures and Tables

**Table 1:** Demographic characteristics of suicide attempters and controls

	*Suicide attempters (*n*=37)*	*Controls (*n*=37)*	*Cohen's* d	P*-value*
Age (years, mean±s.d.)	39±14	38±17	0.06	0.65^a^
Female (%)	70	65	N/A	0.62^b^
BMI (mean±s.d.)	23±5	24±3	0.24	0.80^a^
MADRS score (mean±s.d.; *n*=31)	18±10	N/A	N/A	N/A
mtDNA units per μl plasma pre-DST, median, IQR	23 556, 11 152–58 494	503, 303–930	2.64	2.98E−12^c^
mtDNA units per μl plasma 0800 hours post DST, median, IQR	26 902, 15 371–50 288	683, 356–1111	4.01	1.37E−13^c^
mtDNA units per μl plasma 1500 hours post DST, median, IQR	34 388, 12 591–64 785 (*n*=36)	755, 287–948	2.55	4.58E−12^c^

Abbreviations: BMI, body mass index; DST, dexamethasone suppression test; IQR, interquartile range; MADRS, Montgomery-Åsberg Depression Rating Scale; mtDNA, mitochondrial DNA; N/A, not applicable.

The concentration of mtDNA was skewed and, therefore, log-transformed before calculation of Cohen's *d.* Raw data are given in the table as this is more interpretable than transformed values.

a Student's *t*-test.

b Pearson's *χ*^2^.

c Mann–Whitney *U*-test.

**Table 2:** Correlations (Spearman's Rho correlation coefficients) between free-circulating plasma mtDNA units and post-DST cortisol in suicide attempters

	*mtDNA units, time point 1*	*mtDNA units, time point 2*	*mtDNA units, time point 3*
Post-DST cortisol 0800 hours	0.49*	0.17	0.11
Post-DST cortisol 1500 hours	0.11	0.07	−0.09

Abbreviations: DST, dexamethasone suppression test; mtDNA, mitochondrial DNA.

*P<0.003.
